# Association Between Endocrine Markers, Accumulated Workload, and Fitness Parameters During a Season in Elite Young Soccer Players

**DOI:** 10.3389/fpsyg.2021.702454

**Published:** 2021-08-31

**Authors:** Hadi Nobari, Elena Mainer-Pardos, José Carmelo Adsuar, Juan Manuel Franco-García, Jorge Rojo-Ramos, Marco Antonio Cossio-Bolaños, Luis Urzua Alul, Jorge Pérez-Gómez

**Affiliations:** ^1^Department of Physical Education and Sports, University of Granada, Granada, Spain; ^2^Health, Economy, Motricity and Education Research Group, Faculty of Sport Sciences, University of Extremadura, Cáceres, Spain; ^3^Department of Exercise Physiology, Faculty of Sport Sciences, University of Isfahan, Isfahan, Iran; ^4^Sports Scientist, Sepahan Football Club, Isfahan, Iran; ^5^Health Sciences Faculty, Universidad San Jorge, Zaragoza, Spain; ^6^Universidad Católica del Maule, Talca, Chile; ^7^Escuela de Kinesiología, Facultad de Salud, Universidad Santo Tomás, Santiago, Chile

**Keywords:** training load, growth hormone, performance, testosterone, 1-RM, VO_2max_ (maximal oxygen uptake)

## Abstract

The purpose of this study was to analyze differences between endocrine markers in soccer players, based on playing positions, and correlations between endocrine markers (testosterone, cortisol, growth hormone, and insulin-like growth factor-1), with accumulated workload training and fitness parameters [maximal oxygen uptake (VO_2max_), countermovement jump (CMJ), and isometric maximal strength (1-RM) of the knee for hamstring (ISH) and quadriceps (ISQ) muscles] during early-, mid-, and end-seasons. Twenty-four elite soccer players under 17 participated in this study. The results showed that there was no difference between levels of the endocrine markers among the different positions of the players. Significant correlations were observed between endocrines parameters and fitness performance (ISQ, ISH, VO_2max_, and CMJ). Regression analysis showed that 1-RM and VO_2max_ were the best predictors of endocrine markers. These findings demonstrated that the activity profiles of youth soccer players were not influenced by endocrine markers. Also, it may be assumed that endocrines levels can be used to better explain the physical capacities of this population. Finally, endocrines markers may help to predict changes in 1-RM and VO_2max_.

## Introduction

Soccer is considered a contact sport and demands a wide variety of skills at different intensities. In this regard, soccer players are mainly running kicking, jumping, sprinting, and changing directions. These physical activities require both maximal strength (1-RM) and anaerobic power of the neuromuscular system (Reilly and Williams, 2003). Some of the physical demands for young soccer players during official matches have been reported, with total distances covering 6–8 km, almost 11% of which is covered at 16.1 km·h^−1^ and around 5% is over 19 km·h^−1^. Players perform 81 accelerations (< -1 m.s^−2^) and 82 decelerations (>-1 m.s^−2^), while a range of 651–2,069 body load impacts (Buchheit et al., 2010; Arruda et al., 2015; Vigh-Larsen et al., 2018).

Previous studies have observed the relationship between soccer training load and injuries (Bowen et al., 2017), fitness level of players (Gil-Rey et al., 2015; Nobari et al., 2020b, 2021a), and recovery and fatigue pre/post-match (Coutinho et al., 2015). Internal loads are a combination of biochemical and biomechanical types of stress, while external loads are movements done by the body associated with physical work (Mclaren et al., 2018). It is necessary to know the effects of these loads to understand the stresses placed in the human body during soccer practice. Different methods have been used to train load monitoring in this population, such as rating of perceived exertion (RPE), visual analog scale (Rebelo et al., 2012; Wrigley et al., 2012), acute-chronic workload ratio, and training monotony or training strain (Haddad et al., 2017). These parameters are easy to calculate and could be important for coaches and practitioners to understand weekly session distribution, responses, and accumulated workload training (AWL) during a season (Nobari et al., 2020a,c).

Soccer has positive effects on growth during puberty (Malina et al., 2004). Physical exercise plays an important role in the production and regulation of testosterone (T), cortisol (C), growth hormone (GH), and insulin-like growth factor-1 (IGF-1) (Kanaley et al., 1997; Nobari et al., 2021e). T and C have been reported to respond to metabolic stress associated with fatigue and recovery from soccer-specific exercises (Kraemer et al., 2004; Walker et al., 2010), and their ratio (T:C) has been shown as an indicator of anabolic/catabolic balance, psychophysical stress and fatigue, and information for the adjustment of training loads (Adlercreutz et al., 1986; Papacosta and Nassis, 2011; Nobari et al., 2021d). Additionally, T is quite important in the power performance of youth soccer players (Moreira et al., 2013; Hammami et al., 2017), and it has been noticed that growth in soccer performance has a relationship with growth in plasma T levels and decrease or maintenance of plasma C levels (Hammami et al., 2017; Nobari et al., 2021e). On the other hand, when GH production is suppressed, performance and exercise tolerance are reduced (Schmikli et al., 2012), whereas endurance training increases IGF-1 circulating levels (Maimoun et al., 2004). This information is relevant for coaches and practitioners to analyze the effectiveness of training load. In fact, it allows to make necessary adjustments to maximize training stimuli and adaptations. Finally, there is a lack of studies exploring the critical hormones associated with the cortical-gonadotropic axis on intense training in youth soccer players. Hence, more studies for this population are warranted.

The most important variables for measuring performance in soccer are physical fitness, technical skills, and tactical performance (Abad Robles et al., 2020; Pardos-Mainer et al., 2021). Testing and monitoring of physical performance of players are essential, and they serve many purposes such as comparing between different positions of soccer players, evaluating training effectiveness, preventing overuse injuries, identifying and selecting talents, and monitoring short-/long-term player development. One of the main goals of youth soccer academies is to optimize performance. The isokinetic strength of knee extensor and flexor muscles, and explosive strength in the form of vertical jumping, have been considered to be determinants of optimal performance in soccer, and they have been related to the monitoring of the effects of training, competitive success, and identification of young talented players (Katartzi et al., 2005a,b; Mujika et al., 2009; Nobari et al., 2020b; Pardos-Mainer et al., 2020). In this sense, the physical condition of soccer players can be assessed through several physical fitness tests to measure speed, power, maximal oxygen uptake (VO_2max_), change in direction, agility, and strength (Hoff, 2005; Nobari et al., 2020b). It is not difficult to evaluate the physical fitness of young players; however, two important keys to consider are the reliability and validity of tests and the difference between the adaptations of soccer training and growth-mediated development (Mirkov et al., 2008; Vanttinen et al., 2011; Pardos-Mainer et al., 2019). Research literature has reported that soccer training induces positive hormonal adaptations (Mejri et al., 2005; Hammami et al., 2018). Hammami et al. (2018) observed in under (U) 17 soccer players a significant alteration in hormonal concentrations and physical fitness parameters (i.e., explosive and sprint tests) for two seasons compared with a control group. Mejri et al. (2005) showed that GH levels were highest at the beginning of the season and then reduced but did not have any effect on IGF-I in soccer players U 19. However, coaches and practitioners need more information on these effects to plan their training programs.

Given the importance of physical fitness, training workload, and endocrine biomarkers in soccer performance (Nobari et al., 2021b,f), it is necessary to understand better the concurrent effects of a season on physical and hormonal parameters for better management of training and competitive workloads. Hence, it was hypothesized that hormonal changes would exist among playing positions during a season, and these correlations would show that endocrines parameters are associated with fitness performance. Therefore, this study had 2-fold main objectives: (i) to analyze differences in endocrine markers (T, C, T:C, GH, and IGF-1) in soccer players based on playing positions; (ii) to analyze the correlations between endocrine markers with AWL and fitness parameters (VO_2max_, countermovement jump (CMJ), isometric muscular strength of the knee extensor for hamstring (ISH) and quadriceps (ISQ) muscles) in soccer players during a full season.

## Materials and Methods

### Experimental Approach to the Problem

**Figure 1:**
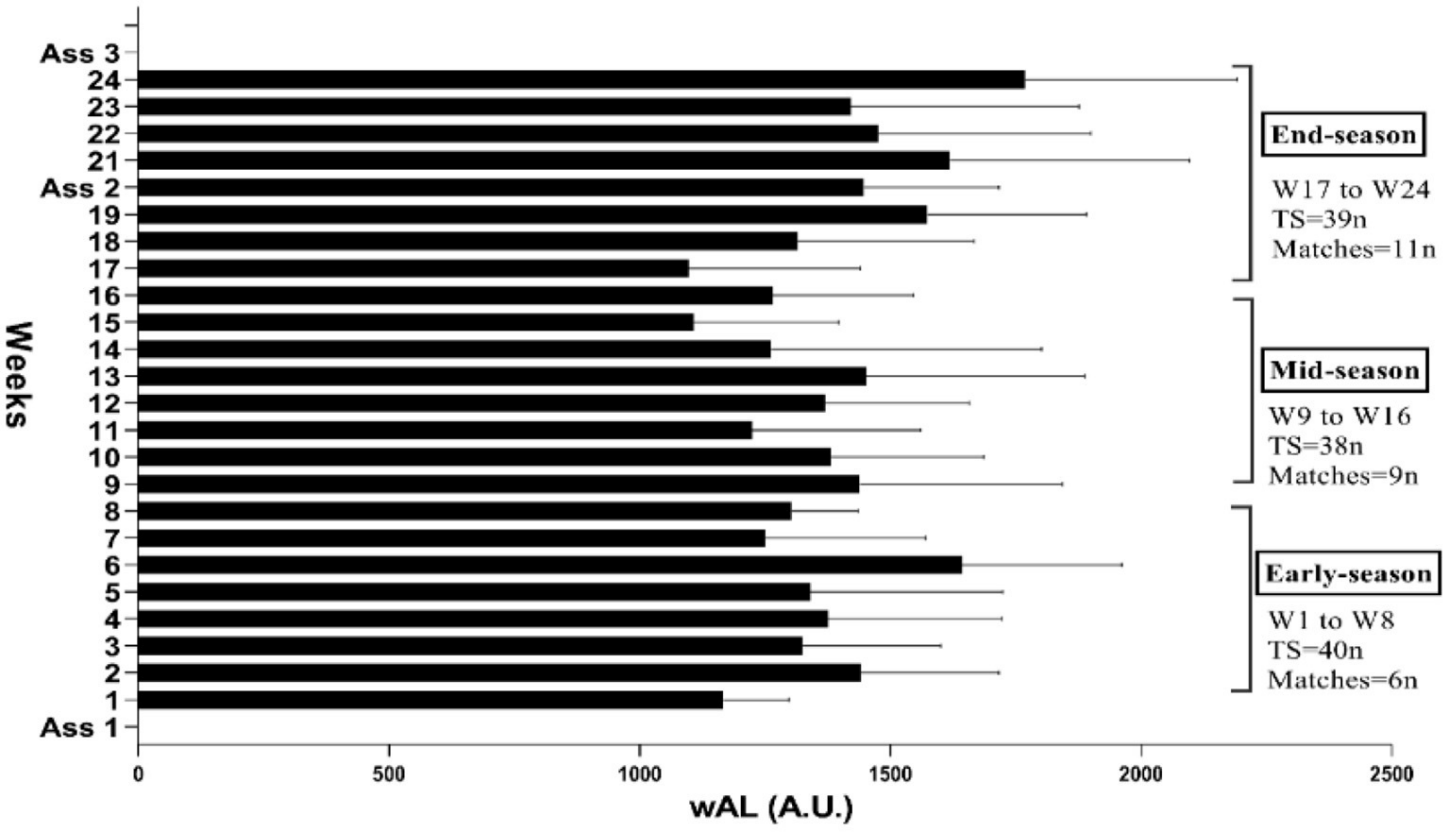
Timeline of monitoring on workload training and evaluations in the entire study. W, week; TS, training sessions; ASS, assessment stages.

A quasi-experimental study with three evaluation stages and a cohort study with 24 weeks of daily training load monitoring was performed on a longitudinal basis, and the results were practical. As can be seen in Figure 1, the season was divided into three periods by week (W) early-season (W1–8), mid-season (W9–16), and end-season (W17–24). The first stage of the evaluation was performed in the first week before the start of the premier league. The second stage took place in the 20th week after the end of the first stage of the premier league, and the third stage took place in the week after the end of the second stage of the premier league final (Figure 1). Anthropometric measurements (pre-season) and hormonal tests (T, C, GH, and IGF-1) were performed only once at the end-season. These measurements were taken in the morning following 12-h fasting, and a minimum 48 h following the last training session (Arazi et al., 2015; Nobari et al., 2020a). Other physical tests were performed separately on a daily basis and in the following order: The CMJ, ISH, and ISQ muscles were done; afterward, the Intermittent Fitness Test 30-15 (30-15_IFT_) for determination of VO_2max_ was conducted. Training sessions and tests were performed in the afternoon. In each session, 30 min after the training (Nobari et al., 2020a), the players were asked to report the RPE, and then the training load was calculated by training time × RPE. Afterward with training load daily AWL was calculated for three periods.

### Participants

Twenty-four elite soccer players under 17 (mean ± standard deviation, SD; age: 16.1 ± 0.22 years; height: 177.6 ± 5.8 cm; body mass: 68.9 ± 7.4 kg; VO_2max_, 46.7 ± 4.28 ml.kg^−1^.min^−1^) participated in the study (Table 1). The players competed in the best premier league in Iran. They were organized based on game positions (Nobari et al., 2021g): three goalkeepers (GK), three forwards (FWs), four center midfielders (CMs), four center halves (CHs), five fullbacks (FBs), and five wingers (WGs). The three inclusion criteria were: each player information is reported in at least 90% of training sessions; they were not allowed to participate in other exercises than team training; players who did not participate in the match each week held a training session to balance training respect to those who played. Before starting, a consent was obtained from the parents and players, and the approval of the ethics committee was obtained from the University of Isfahan and University of Mohaghegh Ardabili. This study complied with the Declarations of Helsinki.

**Table 1:** Description of anthropometric, physical indicators, and accumulated training load of soccer players under 17.

**Demographic**	**Mean ± SD**	**Confidence Interval 95%**		
		**Mean value**	**Lower**	**Upper**
Age (years)	16.1 ± 0.2	0.1	16.0	16.2
Height (cm)	177.6 ± 5.8	2.3	175.3	179.9
Weight (kg)	68.9 ± 7.4	3.0	65.9	71.8
BMI (kg.m^2^)	21.8 ± 1.8	0.7	21.1	22.5
VO_2max_ (ml.kg^−1^.min^−1^)	46.7 ± 4.3	1.7	45.0	48.4
ISH (Nm)	78.8 ± 19.6	7.8	70.9	86.6
ISQ (Nm)	205.9 ± 73.6	29.4	176.5	235.3
CMJ (cm)	41.8 ± 4.1	1.6	40.1	43.4
AWL (AU)	31215.7 ± 5244.9	2098.4	29117.3	33314.0

*SD, Standard deviation; BMI, body mass index; VO_2max_, maximal oxygen consumption; ISH, isometric muscular strength of the hamstring muscles; ISQ, isometric muscular strength of the quadriceps muscles; CMJ, countermovement jump; Nm, newton meter. AWL, accumulated workload training; AU, arbitrary unit*.

### Sample Size

Various studies have shown a large to very large association of fitness parameters with training load (Nobari et al., 2020a,c) and endocrine markers (Mejri et al., 2005; Hammami et al., 2018; Perroni et al., 2020) in young soccer players. Therefore, the results were analyzed to obtain a sample size with at least 0.9 power. The variables considered in the analysis were two-tailed, α error probability 0.05, and large to very large association.

### Data Measurement and Variables

#### Anthropometric Measurements

Height was measured with a Seca 213 (Seca, Hamburg, Germany) with an accuracy of ±5 mm, and for body mass a Seca 813 (Seca, Birmingham, United Kingdom) with an accuracy of 0.1 per kg was used. The measurements were followed according to the recommendation of the International Society for the Advancement of Kinanthropometry (ISAK) standards (Norton and Olds, 1996), and performed in the morning before breakfast (Arazi et al., 2015; Ali Jafari et al., 2016). To measure sitting height as in a previous study (Nobari et al., 2020a,c), the subjects were asked to sit on a box 50 cm high, facing forward. The outcomes obtained were height in cm and body mass in kg.

##### Blood Analysis

Measurements of testosterone, cortisol, growth hormone, and insulin-like growth factor-1 were performed in the laboratory ward of Al-Zahra Hospital. All the players went to the laboratory after 12 h of fasting, and 2 days after the last training session of the team, at eight o'clock in the morning. As in the previous study process (Nobari et al., 2021c), 10 cc of blood was first taken from the antecubital vein of the players by a qualified phlebotomist. The collected samples were immediately centrifuged, and their serum was removed. To measure GH and IGF1, the chemiluminescence method (ICMA) with IMMULITE 2000 XPi System (Siemens, Munich Germany) was used. The analytical sensitivity of the kit is 0.01 ng/ml. The average inter-assay coefficients of variability (CVs) were 3.76%, and mean concentrations were 7.3 ng/ml. For IGF-1, the limit was 13.3 ng/ml. The average inter-assay CVs were 4.4%, and the mean concentration was 379.83 ng/ml. To measure T and C, the ICMA with ADVIA Centaure XPT System (Siemens, Munich, Germany) was used, and the analytical sensitivity of assay ranges was 0.20–75 μg/dl (5.5–2,069 nmol/L). The average inter- and intra- assay CVs were 3.29 and 3.64%, respectively, for the mean concentration of 17.67 μg/dl. For T, the analytical sensitivity of assay ranges was 10–1,500 ng/dl (0.35–52.1 nmol/L). The average inter- and intra- assay CVs were 4.05 and 3.7%, respectively, for the mean concentration of 567.17 ng/ml. The ratio T:C was also calculated.

##### Countermovement Jump

Countermovement jump was used to measure the explosive power of the lower body (Bangsbo and Mohr, 2012). A Finnish-made device called Newtest Powertimer SW-300 (Newtest Oy, Oulu, Finland) was used. Before starting, each player warmed up for 10–15 min under the supervision of a strength and conditioning coach, which included jumping-like (e.g., CMJ, horizontal bounds, and vertical hops) movements (Nobari et al., 2020c). They also performed two jumps on-screen tests for familiarity. The players stood on the screen, and a model without arm-swing movements was used for testing. They bent the knee up to about 90° and then jumped to maximum power with commands of the tester. Each player performed two repetitions, with a 5-min break, and records were recorded in cm (Haugen et al., 2012). The best record was considered as a criterion for analysis. The intra-class correlation (ICC) was 0.94 in the CMJ.

##### Knee Flexor and Extensor Isometric Tests

To measure isometric muscular strength of the knee extensor for hamstring and quadriceps, three repetitions, respectively, were performed, forward and backward forces for 5-s (10-s rest between repetition and 2-min rest between change type of contraction) in a standard isokinetic Biodex (Shirley, NY, United States) system model-3, which has been reported to have high validity in assessing net peak torque (Drouin et al., 2004). All the participants were tested by the dominant leg, selected based on the leg that is used to kick in the games (Daneshjoo et al., 2012). They familiarized the test and warmed up with a Monark cycle ergometer (COSMED, Concord, CA, United States). Then, five flexion and extension trials at 90° were performed. The Biodex device was calibrated according to its handbook before the test commenced. The dynamometer seat was fixed at a 90° angle, and the back seat angle was set at 70–85° that the participant felt comfortable. The sitting zone was set in which knee could easily be moved and quadriceps force made extension. The rotation axis of the knees (lateral femoral epicondyle) was aligned with the dynamometer mechanical axis. To measure the strength of related knee muscles, in seated position. The body, waist, and femur of participants were fixed with special tape to the seat. The best result of three maximal force trials were recorded as performance of isometric peak torque (Nm) (Steffen et al., 2008). If there were 10% higher peak torque differences in the three trials, then an additional trial was performed (Alvares et al., 2015). Output force ratio of the hamstring to quadriceps (H:Q) was also calculated for balance of agonist and antagonist of relevant knee muscles (Aagaard et al., 1998). All the participants were encouraged orally by the researcher to perform the tests better. The test retest was performed to calculate ICC, which was 0.94 for this test.

##### Intermittent Fitness Test 30-15

Intermittent fitness test 30-15 was used for maximal oxygen uptake, and shuttle run tests were performed in the field; alternative recovery test consists of repeated 2 × 20-m runs forward and back between the starting, turning, and finishing lines (equals 40-m shuttle) at a progressively increased speed controlled (0.5 km.h^−1^ in each stage) with a beep sound; the average speed started at 8 km.h^−1^ (Buchheit, 2010). After the first beep, the athlete starts running at 8 km. h^−1^ for the 30-s bout. Between each running bout, they have a 15 s rest period, until exhaustion. Each participant in the repetition who could not do another repetition or did not reach the finish line three times was recorded as an individual record. Then, the following formula was used to estimate the VO_2max_ = 28.3 – (2.15 × 1) – (0.741 × 17 years) – (0.0357 × body mass) + (0.0586 × 17 years × VIFT) + (1.03 × VIFT), where VIFT = the final speed of the subjects in the test exhaustion. Before the test, all the athletes warmed up under the supervision of a strength and conditioning coach. The test-retest was performed to calculate ICC, which was 0.92 for this test.

##### Monitoring Internal Training Loads

The players were monitored daily for their perceived exertion using CR-10 Borg's scale, which is valid and reliable for estimation of the intensity of a session (Foster et al., 2001). To the question “How intense was your session?”, the players answered in the interval of 0 (minimum effort) and 10 (maximal effort). They answered using the scale, 30 min after the end of training session. Additionally, the duration of the training sessions (in minutes) was recorded. As a measure of internal load, the session-RPE was calculated by multiplying the score on the CR-10 scale by the duration of the session in min. The subjects were previously familiarized with the scale, using it in the previous 2 years in the club. In this study, the AWL was used for 24 weeks. These weeks of the full-competition season were divided into three periods (early-season, W1–W8; mid-season, W9–W16; and end-season, W17–W24). The outcome was expressed in arbitrary units (AU).

### Statistical Analysis

Statistical analyses were conducted using the two software; (i) Graph-Pad Prism 8.0.1, and (ii) Statistical Product and Service Solutions (SPSS, version 23.0). We removed the training load monitoring information before the first phase assessments in this study. A significance level of *p* < 0.05 was the criterion in all the analyses. Shapiro–Wilk was used for considering the criterion normality of the data. The steps of inferential statistical implementation were performed as follows. First, a one-way analysis of variance (ANOVA) was performed to compare the endocrine markers by playing position. Afterward, Pearson correlation analysis was performed between IGF-1, C, and physical performance tests, and Spearman correlations were performed for GH, T, T:C ratio, Q:H ratio, and AWL periods owing to non-normality. The thresholds of correlation (r) were defined as (Hopkins et al., 2009): < 0.1 = trivial;0.1–0.3 = small;0.3–0.5 = moderate;0.5–0.7 = large;0.7–0.9 = very large; and >0.9 = nearly perfect. Consequently, linear regression was used to predict the variables aforementioned with the endocrine markers due to the good correlation results in them. The G-Power software (University of Dusseldorf, Dusseldorf, Germany) was used to perform statistical population calculation. The model used was *a priori*, considered in accordance with the main purpose of the study: *t*-tests - correlation: Point biserial model.

## Results

**Figure 2:**
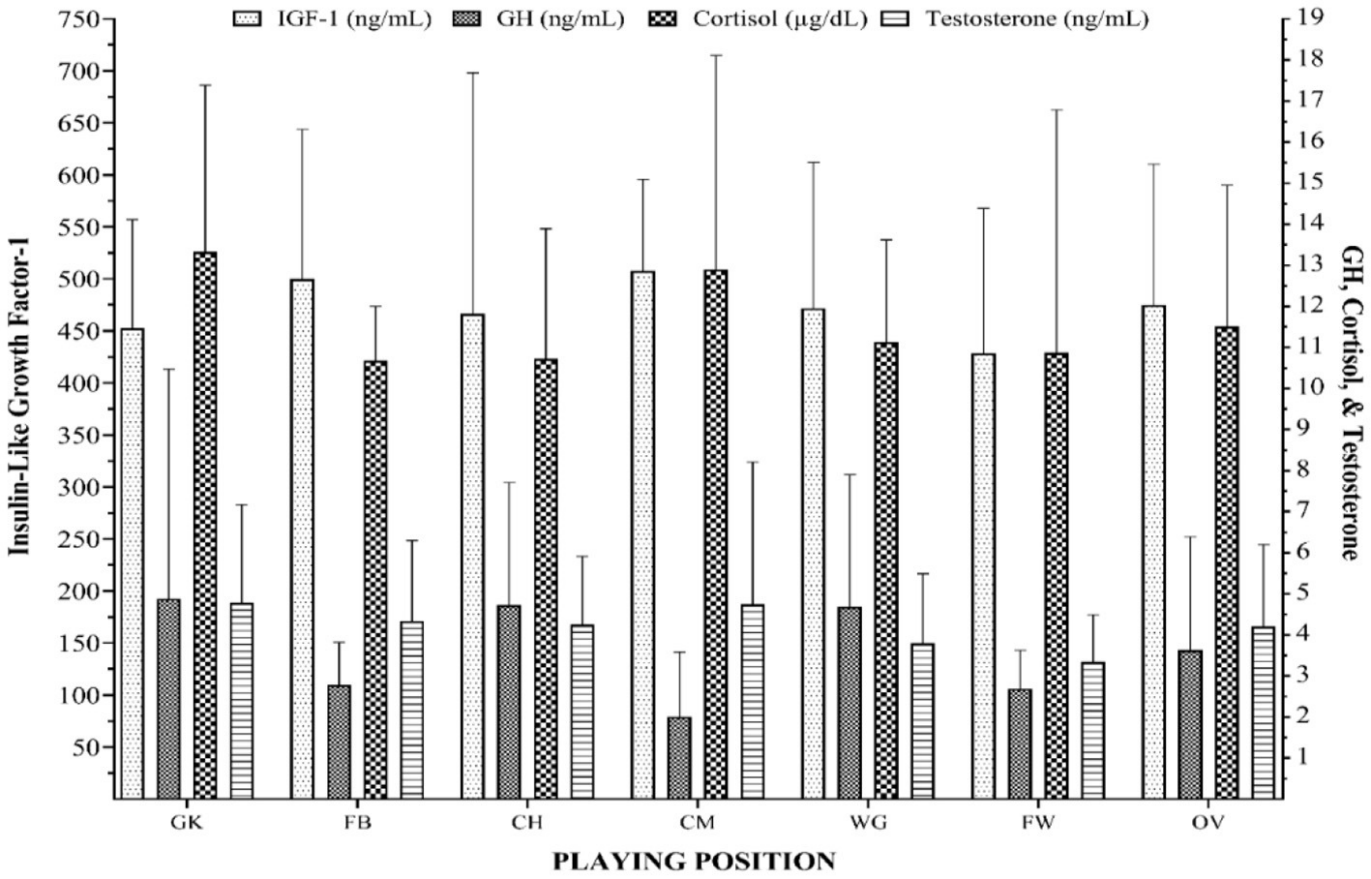
Levels of endocrine markers of soccer players according to playing position and the overall of team after matches of season. GK, goalkeepers; FW, forwards; CM, center-midfielder; CH, center-half; FB, fullback; WG, winger; IGF-1, insulin-like growth factor-1; GH, growth hormone.

Figure 2 demonstrates growth hormone, insulin-like growth factor-1, cortisol, and testosterone levels based on the position of the players under 17. The results of one-way ANOVA showed that there was no difference in endocrine markers among the different positions of the players.

Table 2 shows relevant associations between endocrine markers, fitness levels, and accumulated workload training. The most important results, with 95% confidence interval (CI 95%) of r, were reported. Pre-season ISQ was significant, moderately to largely correlated with all hormones. VO_2max_ during the mid-season was moderately associated with GH [r = 0.49; CI 95% = 0.08–0.75*;p* = 0.02], largely associated with IGF-1 [r = 0.52; CI 95% = 0.15–0.76; *p* = 0.009], and moderately correlated with CMJ [r = 0.41; CI 95%= −0.01–0.71*;p* = 0.04].C showed a moderate-to-large negative correlation with VO_2max_ [r = −0.55; CI 95%= −0.78 to −0.19; *p* = 0.006] and CMJ [r = −0.46; CI 95%= −0.73 to −0.07*;p* = 0.02] during mid-season.

**Table 2:** Associations between endocrine markers and fitness variables, and accumulated workload training.

**Variable**	**β0**	**β1**	**β2**	**β3**	**β4**	**β5**	**β6**	**β7**	**β8**	**β9**	**β10**	**β11**	**β12**	**β13**	**β14**	**β15**	**β16**	**β17**	**β18**	**β19**	**β20**	**β21**	**β22**
IGF-1 (β0)	1																						
GH (β1)	**0.65**	1																					
Cortisol (β2)	**−0.55**	**−0.66**	1																				
Testosterone (β3)	**0.55**	**0.64**	**−0.48**	1																			
T:C (β4)	**0.67**	**0.72**	**−0.79**	**0.89**	1																		
ISQ1 (β5)	**0.64**	**0.68**	**−0.50**	**0.39**	**0.49**	1																	
ISQ2 (β6)	**0.62**	**0.69**	**−0.48**	**0.39**	**0.48**	**0.99**	1																
ISQ3 (β7)	**0.62**	**0.71**	**−0.55**	**0.40**	**0.51**	**0.96**	**0.94**	1															
ISH1 (β8)	0.32	**0.61**	−0.34	0.21	0.31	**0.76**	**0.74**	**0.73**	1														
ISH2 (β9)	**0.52**	**0.60**	−0.35	0.37	**0.39**	**0.75**	**0.78**	**0.74**	**0.71**	1													
ISH3 (β10)	0.37	**0.55**	−0.31	0.27	0.33	**0.58**	**0.55**	**0.67**	**0.80**	**0.71**	1												
H:Q1 (β11)	**−0.61**	**−0.39**	0.37	−0.25	−0.33	**−0.73**	**−0.72**	**−0.73**	−0.07	**−0.39**	0.03	1											
H:Q2 (β12)	**−0.47**	**−0.46**	**0.41**	−0.20	−0.33	**−0.78**	**−0.75**	**−0.77**	−0.32	−0.22	−0.03	**0.81**	1										
H:Q3 (β13)	−0.38	−0.29	0.29	−0.13	−0.21	**−0.58**	**−0.56**	**−0.59**	0.02	−0.11	0.32	**0.83**	**0.87**	1									
VO_2max_1 (β14)	**0.49**	**0.50**	**−0.46**	0.24	0.34	**0.59**	**0.55**	**0.54**	0.38	0.33	0.16	0.36	0.27	**−0.48**	1								
VO_2max_2 (β15)	**0.52**	**0.49**	**−0.55**	0.23	0.37	**0.62**	**0.59**	**0.62**	**0.44**	**0.48**	0.34	**0.46**	0.37	**−0.45**	**0.93**	1							
VO_2max_3 (β16)	**0.44**	**0.45**	−0.38	0.20	0.25	**0.58**	**0.55**	**0.58**	0.37	**0.41**	0.23	**0.39**	0.24	**−0.47**	**0.93**	**0.93**	1						
CMJ1 (β17)	0.32	0.38	−0.26	0.00	0.13	**0.44**	**0.42**	**0.43**	0.18	0.19	0.14	0.17	0.14	**−0.49**	0.33	0.28	0.36	1					
CMJ2 (β18)	**0.47**	0.52	**−0.46**	0.15	0.32	**0.47**	**0.45**	**0.47**	0.29	0.26	0.33	0.27	0.34	**−0.41**	0.39	0.41	**0.42**	**0.86**	1				
CMJ3 (β19)	**0.43**	0.35	−0.37	0.09	0.26	0.31	0.29	0.33	0.14	0.15	0.17	0.04	0.11	−0.33	0.31	0.29	0.31	**0.78**	**0.76**	1			
EarS AWL (β20)	0.12	−0.15	−0.04	−0.06	0.00	−0.12	−0.16	−0.20	**−0.44**	−0.04	**−0.47**	−0.11	0.00	−0.19	−0.09	0.00	−0.06	−0.02	−0.12	−0.16	1		
MidS AWL (β21)	0.10	−0.18	−0.06	−0.24	−0.08	0.03	0.03	0.00	−0.25	0.03	−0.29	0.13	−0.31	−0.26	0.11	0.14	0.10	0.05	−0.06	−0.17	**0.75**	1	
EndS AWL (β22)	0.15	−0.09	0.02	−0.11	−0.09	0.13	0.13	0.10	−0.31	0.15	**−0.43**	**−0.43**	−0.26	**−0.49**	0.19	0.20	0.25	0.31	0.19	0.16	**0.65**	**0.61**	1

*Significant differences (p ≤ 0.05) are highlighted in bold. IGF-1, insulin-like growth factor-1; GH, growth hormone; T:C, ratio, testosterone to cortisol; ISH, isometric muscular strength of the hamstring muscles; ISQ, isometric muscular strength of the quadriceps muscles; H:Q, the strength of isometric hamstring to quadriceps ratio; VO_2max_, maximal oxygen uptake; CMJ, countermovement jump; AWL, accumulated workload training; EarS AWL, the average of AWL in early-season; MidS AWL, the average of AWL in mid-season; EndS AWL, the average of AWL in end-season*.

**Figure 3:**
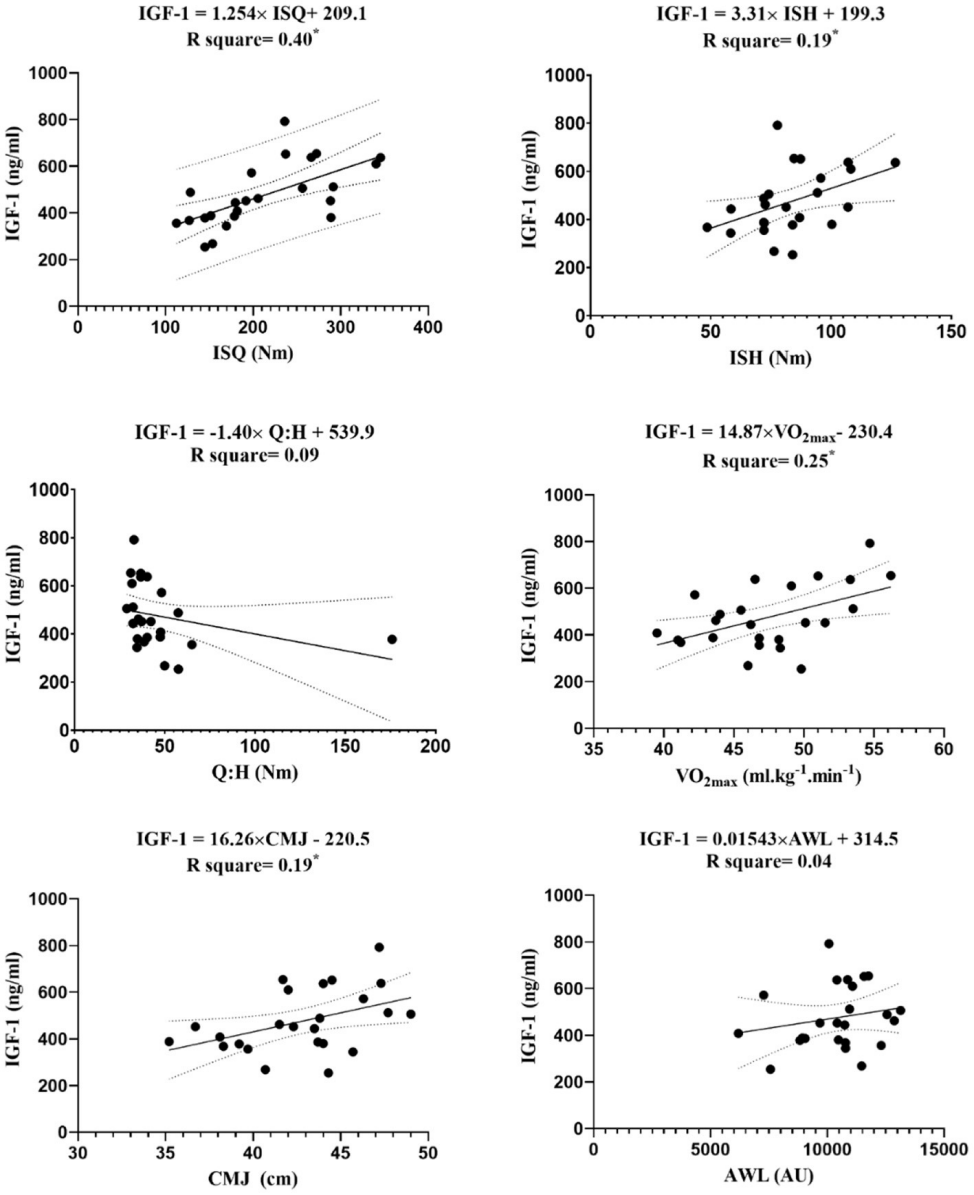
Regression analysis to explain fitness levels and AWL (the average of the three stages) with IGF-1, insulin-like growth factor-1; ISH, isometric muscular strength of the hamstring muscles; ISQ, isometric muscular strength of the quadriceps muscles; H:Q, the strength of isometric quadriceps to hamstring ratio; VO_2max_, maximal oxygen uptake; CMJ, countermovement jump; AWL, accumulated workload; Nm, Newton meter; AU, arbitrary unit. *Significant differences *p* ≤ 0.05.

**Figure 4:**
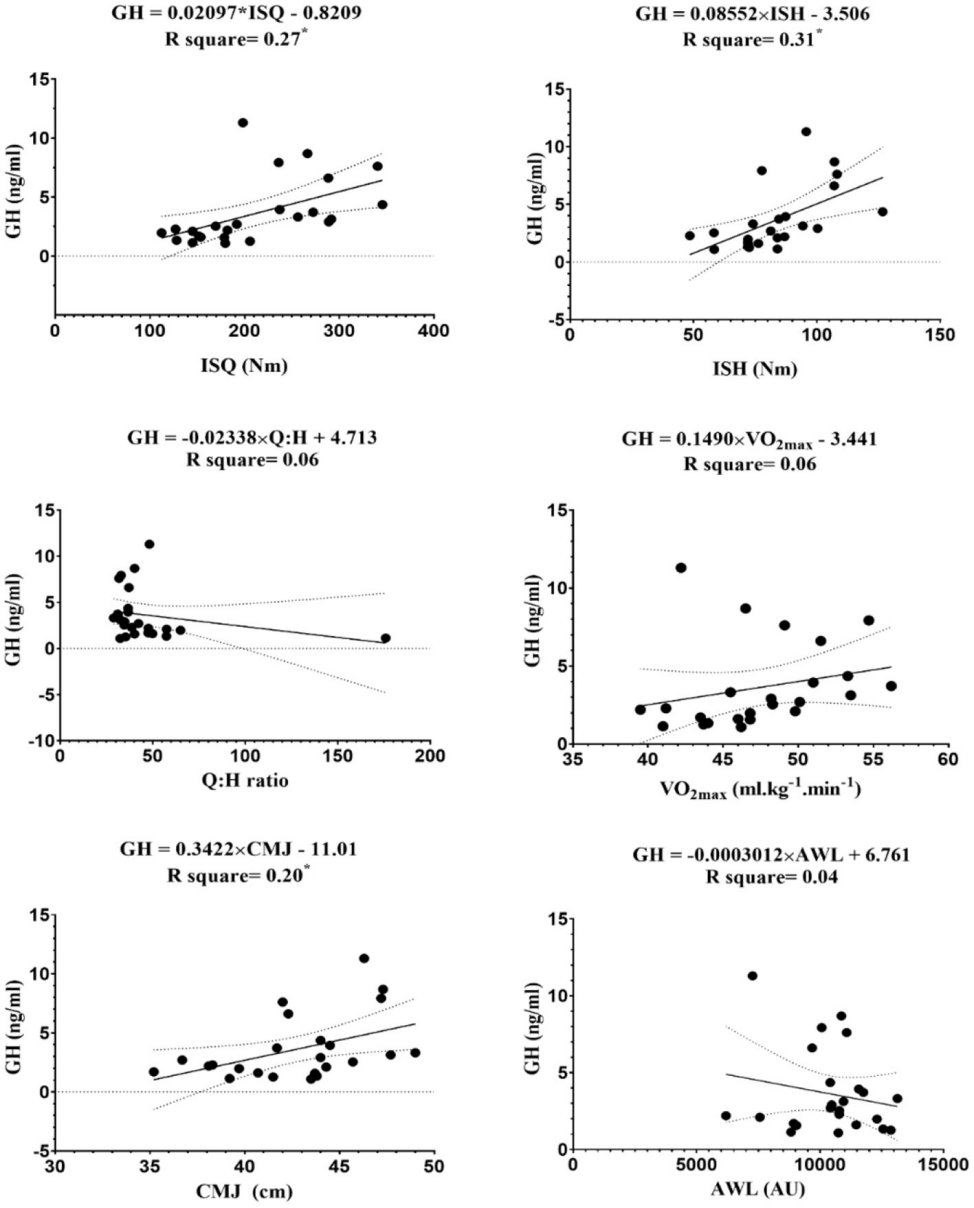
Regression analysis to explain fitness levels and accumulated workload training (AWL) (the average of the three stages) with GH, growth hormone; ISH, isometric muscular strength of the hamstring muscles; ISQ, isometric muscular strength of the quadriceps muscles; H:Q, the strength of isometric quadriceps to hamstring ratio; VO_2max_, maximal oxygen uptake; CMJ, countermovement jump; AWL, accumulated workload training; Nm, Newton meter; AU, arbitrary unit. *Significant differences *p* ≤ 0.05.

**Figure 5:**
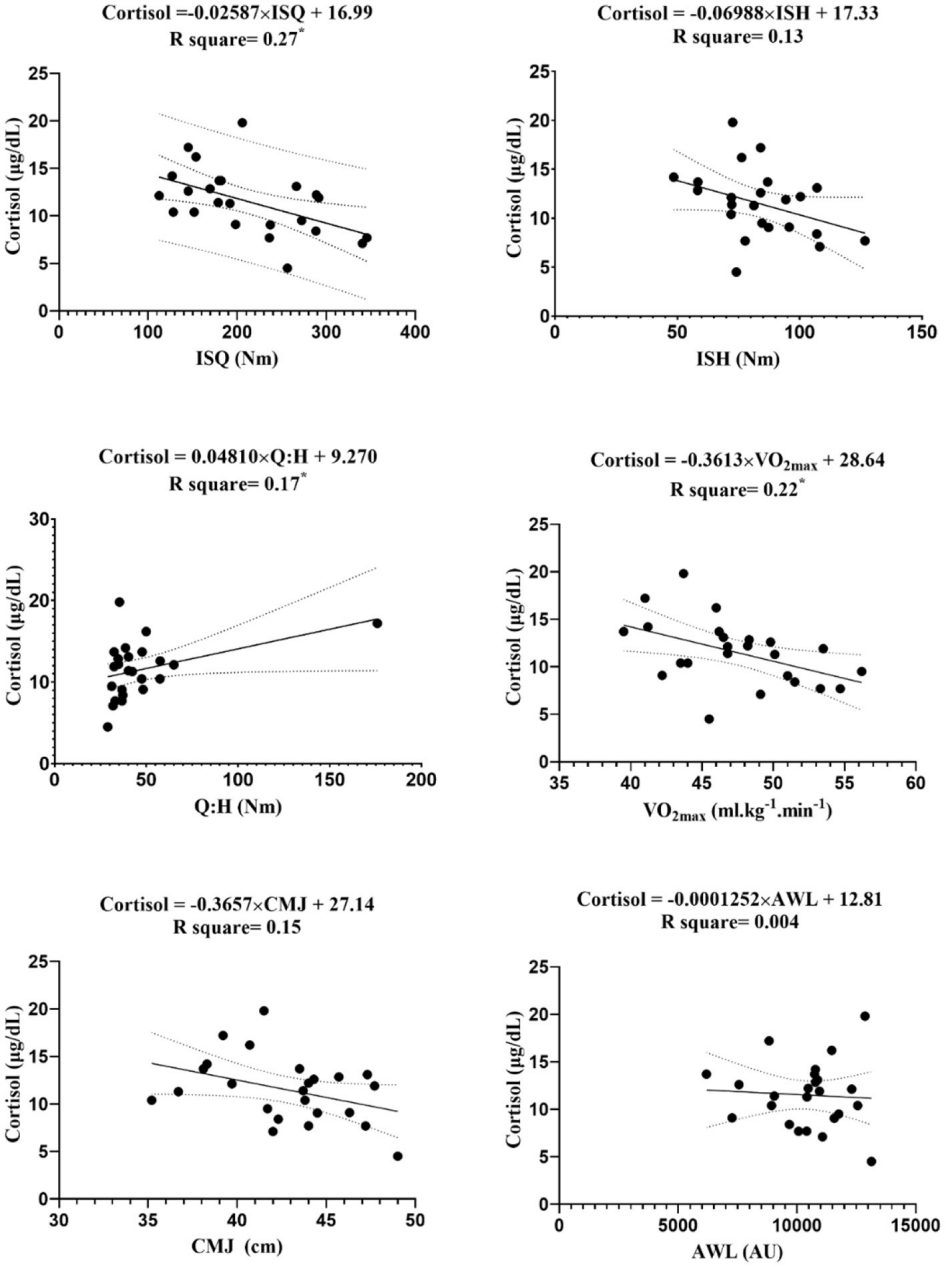
Regression analysis to explain fitness levels and AWL (the average of the three stages) with cortisol; ISH, isometric muscular strength of the hamstring muscles; ISQ, isometric muscular strength of the quadriceps muscles; H:Q, the strength of isometric quadriceps to hamstring ratio; VO_2max_, maximal oxygen uptake; CMJ, countermovement jump; AWL, accumulated workload training; Nm, Newton meter; AU, arbitrary unit. *Significant differences *p* ≤ 0.05.

**Figure 6:**
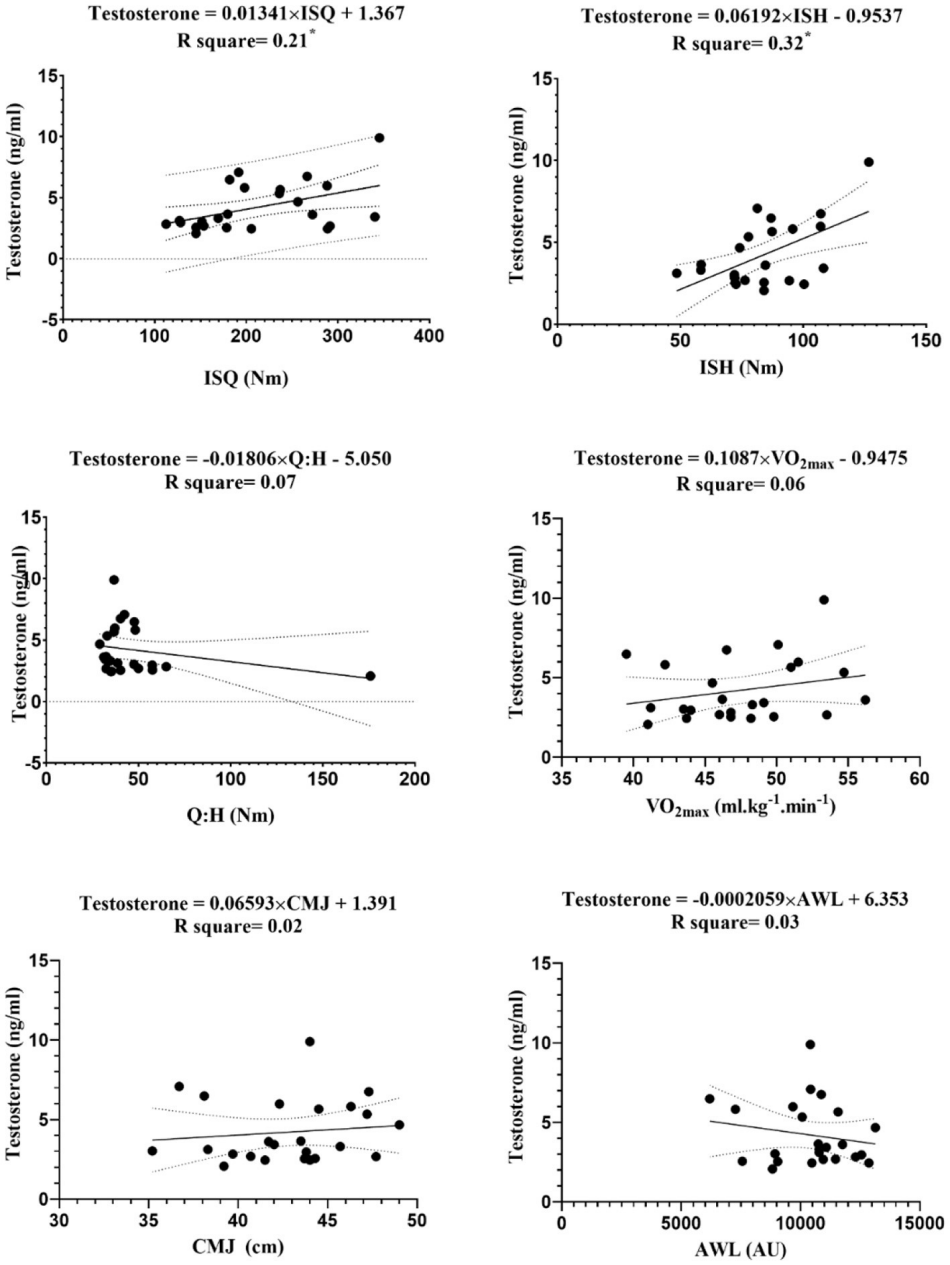
Regression analysis to explain fitness levels and AWL (the average of the three stages) with testosterone; ISH, isometric muscular strength of the hamstring muscles; ISQ, isometric muscular strength of the quadriceps muscles; H:Q, the strength of isometric quadriceps to hamstring ratio; VO_2max_, maximal oxygen uptake; CMJ, countermovement jump; AWL, accumulated workload training; Nm, Newton meter; AU, arbitrary unit. *Significant differences *p* ≤ 0.05.

**Figure 7:**
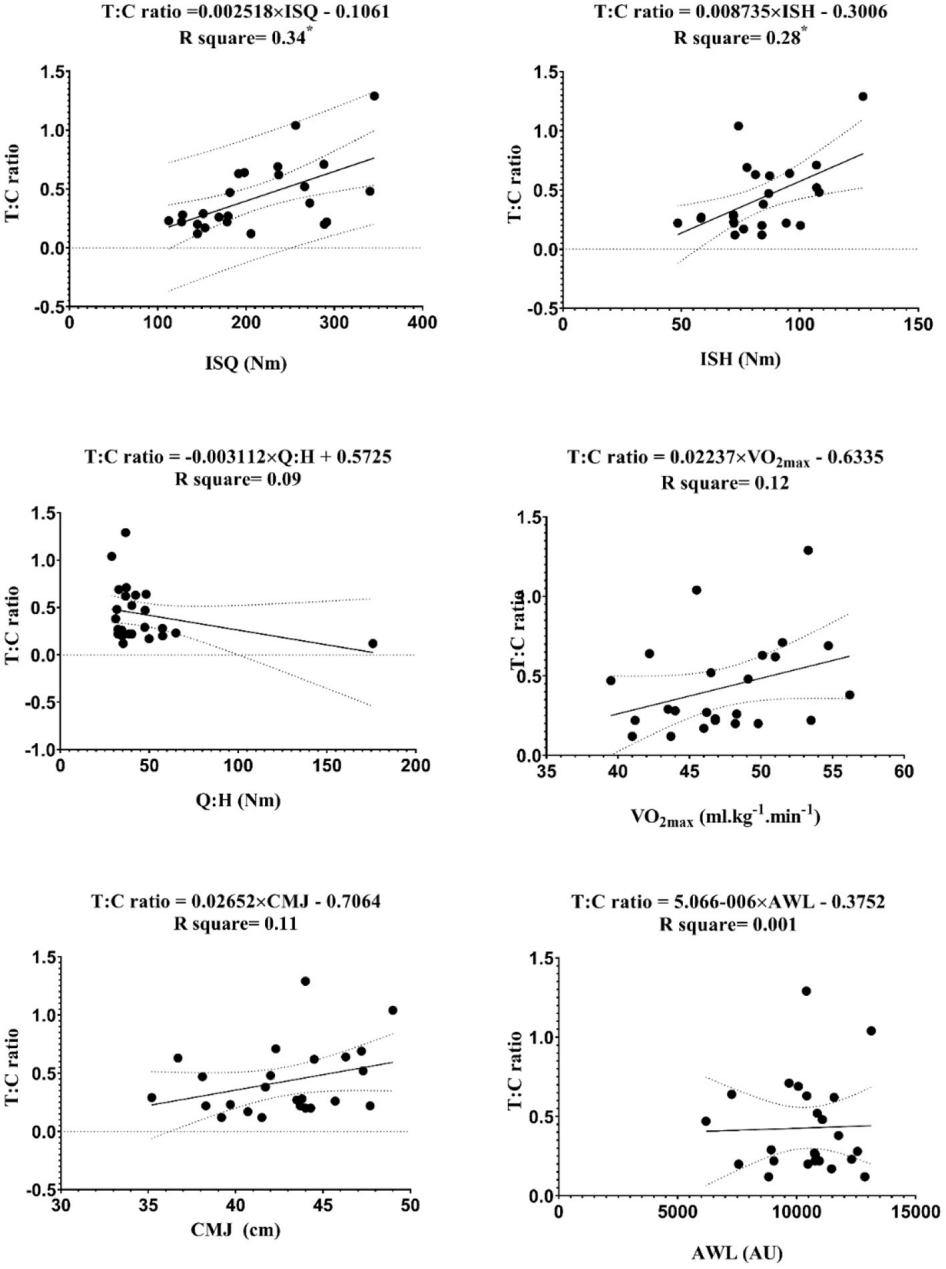
Regression analysis to explain fitness levels and AWL (the average of the three stages) with GH, growth hormone; ISH, isometric muscular strength of the hamstring muscles; ISQ, isometric muscular strength of the quadriceps muscles; H:Q, the strength of isometric quadriceps to hamstring ratio; VO_2max_, maximal oxygen uptake; CMJ, countermovement jump; AWL, accumulated workload training; Nm, Newton meter; AU, arbitrary unit. *Significant differences *p* ≤ 0.05.

The linear regression between endocrine markers with independent variables (i.e., ISQ, ISH, Q:H ratio, VO_2max_, CMJ, and AWL) are reported in Figures 3–7.

The figures show that the equations derived from all the hormones are good predictors of isometric maximal strength variables. Moreover, the results of this study demonstrate that IGF-1 can significantly predict the performance of CMJ [*F*_(1, 22)_ = 5.12, β = 16.26, *p* = 0.03] with an R^2^ = 0.19, and VO_2max_ [*F*_(1, 22)_ = 7.15, β = 14.87, *p* = 0.01] with an R^2^ = 0.25. The levels of IGF-1 increased by 16.26 ng/ml for each cm of CMJ, and −14.87 ng/ml for each ml.kg^−1^.min^−1^ of VO_2max_. The obtained equation GH was [*F*_(1, 22)_ = 5.53, β = 0.34, *p* = 0.03] with an R^2^ = 0.2, so GH can be a good predictive power for CMJ. Ultimately, the obtained equation hormone C was [*F*_(1, 22)_ = 6.36, β = −0.36, *p* = 0.02] with an R^2^ = 0.2, so hormone C can be a proper predictive power for VO_2max_.

## Discussion

The goal of this study was to analyze differences between endocrine markers in soccer players, based on playing positions, and correlations between endocrine markers with AWL and fitness parameters based on playing positions. The main findings of this study were that IGF-1 and GH were positively correlated with ISQ, ISH, VO_2max_, and CMJ in a moderate to large magnitude, and negatively correlated with C, with same variables and magnitude. In addition, T and T:C ratio were moderately correlated with ISQ. The linear regression model, using the status of endocrine markers, fitness status, and AWL, explained that it obtained good predictions from the variables 1-RM (e.g., ISQ and ISH) and VO_2max_. The endocrine markers were not able to determine AWL by any of the models.

Young players of different positions have a very different workload during a game. FWs had the best performances in endurance, velocity, agility, and power, while GKs had the worst performance in these parameters (Gil et al., 2007). It is well-known that the role of GKs is very different from that of other players. Consequently, their demands and training are diverse from other playing positions on a soccer team. Taking into account the endocrine results, IGF-1 levels in the FBs and CMs were higher than the rest of the player positions. In CH and WG, we observed an increase in GH levels. Finally, GK and CM showed that C and T concentrations were higher than in other positions. However, no statistical differences were found between the levels of endocrine parameters and the positions of players. To the best knowledge of the authors, this is the first study to monitor endocrine markers during a soccer season in different youth soccer player positions, and further studies are required to establish stronger conclusions.

It is well-known that physical exercise plays a significant role in the regulation of the GH and IGH-1 axis (Kanaley et al., 1997). The results showed significant correlations between GH and IGF-1, and the fitness parameters (ISQ, ISH, VO_2max_, and CMJ). During and after exercise, a number of stimuli affect GH release, such as the duration and intensity of exercise, lactate, core temperature, or environmental factors (i.e., ambient temperature) (Langfort et al., 2001; Wheldon et al., 2006; Nobari et al., 2021e). Similar to the results, Eliakim et al. (2010) found a positive association between fitness parameters and overnight GH levels in adolescent females. In contrast, GH levels were not identified to be correlated with physical performance in U17 soccer players (Hammami et al., 2018). Therefore, findings in the scientific literature are controversial, and maybe the GH response to training is related with the intensity of training, sex, age, and physical activity practiced.

Exercise stimulates growth hormone release, and growth hormone induces the production of insulin-like growth factor-1 and its increase in plasma (Pavelic et al., 2007). Previous research studies have found a positive correlation between physical fitness and concentration levels of IGF-1 in a youth population (Brun et al., 1996; Hammami et al., 2018). Brun et al. (1996) observed that IGF-1 positively correlated with aerobic performance in children, while Hammami et al. (2018) showed a significant association among IGF-1 and CMJ, squat jump, and five consecutive long jumps in young soccer players. These results agree with those of this study, where IGF-1 levels were positively correlated with fitness parameters. Thus, this positive correlation demonstrates that high intensity exercise is related with increased activity of the IGF-1 system benefiting an anabolic state (Eliakim et al., 1998). Thus, this increase in IGF-1 has a practical value for coaches. This means an endocrine adjustment to training and physical anticipation of further exercise. T and C are reliable markers of training stress that observe the anabolic/catabolic state of the body in response to exercise (Kraemer et al., 2004). Over-reaching/over-training can be monitored by measuring C and, consequently, excessive stress in athletes can induce performance reduction (Kraemer et al., 2004; Hammami et al., 2018). In this study, significant inverse correlations were observed between C and fitness parameters (ISQ, ISH, VO_2max_, and CMJ). It indicates that those subjects with a larger decrease in C may be more likely to produce major isometric and explosive strength and endurance gains than those with smaller decreases in C. Kraemer et al. (1998) have reported that a decrease in circulating C after training reflects a decreased level of tissue muscle breakdown and contributes to the overall enhancement of the anabolic environment. Thus, this inverse correlation between C and fitness parameters could suggest that lower C levels improve soccer performance. Hence, the data indicate that it is important to control endocrines markers to well-design training loads throughout a soccer season and consequently to prevent overuse injuries and to improve fitness performance.

T enhances the neural adaptations to repair and recover muscle and to gain physical and capacities performance (Kraemer and Ratamess, 2005). T:C represents the anabolic and catabolic activity, and indicates the general stress of training and the early identification of an imbalance between catabolic and anabolic metabolism (Adlercreutz et al., 1986). The results showed significant positive correlations between T and T:C ratio with ISQ. It indicates that young soccer players with an increase in T and T:C ratio may be more likely to produce major isometric quadriceps strength gains than those with smaller decreases in these parameters. These data were in line with other studies that demonstrated a decrease in T and T:C ratio was associated with a decrease in performance (Adlercreutz et al., 1986; Crewther et al., 2006). Nevertheless, prospective studies should monitor the response of these parameters to long-term training loads to assist coaches in controlling the training process, improving the performance of players, and stimulating their maximal physiological adaptation without inducing overtraining. It was found that endocrine markers were good variables to predict 1-RM (e.g., ISQ and ISH) and VO_2max_ performance. Endocrine markers are acknowledged to be important factors in performance (Adlercreutz et al., 1986; Crewther et al., 2006; Hammami et al., 2018). As discussed previously, it has been observed that a catabolic state can diminish force production, caused by losses of contractile proteins or neural transmitters usually stimulated by T interactions and, due to mechanisms, may decrease isokinetic strength (Florini, 1987). The decrease in the ability to produce force may be related to performance reductions in strength and, consequently, endurance. The data from the investigation indicate a decrease catabolic environment within the muscles, which has contributed to increased knee extensor and flexor strength and endurance performance. This may help to explain the results.

Finally, in the process of identifying talent and avoiding overtraining in this period, the importance of measuring endocrine markers and physical performance during adolescence needs to be emphasized, because endocrine markers are correlated with performance, and they could help to control training loads.

This study has some limitations. First, the blood samples were taken at the end-season, which does not allow to report adaptations to training at different moments of the season. Second, young soccer players of the same category and chronological age were analyzed. For these reasons, further research studies should examine additional hormonal and physical markers, in similar or different populations compared with a control group, to further elucidate this research topic and provide more insights.

## Conclusions

This research reveals associations between endocrines parameters and fitness performance (ISQ, ISH, VO_2max_, and CMJ). However, the activity profiles of young soccer players were not influenced by endocrine markers. Finally, 1-RM and VO_2max_ were the best predictors of endocrine markers. Thus, it is recommended that coaches and practitioners should consider endocrine parameters and fitness performance during a soccer season to control training loads. Future research is warranted to observe the relationship between different endocrine parameters (i.e. IGFBP-3, erythropoietin, or estrogen for female athletes), genes related to performance such as angiotensin-1 converting enzyme insertion/deletion (ACE I/D) polymorphism or α-actinin-3 (ACTN3) R577X polymorphism (Guth and Roth, 2013), and fitness performance with different training intensities, ages, or sex in soccer players.

## Data Availability Statement

The original contributions presented in the study are included in the article/Supplementary Material, further inquiries can be directed to the corresponding author.

## Ethics Statement

Before starting of this study, consent form was obtained from parents and players and the approval of the ethics committee was obtained from the University of Isfahan and University of Mohaghegh Ardabili. This study complied with all declarations of Helsinki.

## Author Contributions

HN and JA: conceptualization, formal analysis, and software. HN, EMP, JA, and JPG: methodology. HN, JA, EMP, and JPG: writing–original draft preparation. HN, JA, and JPG: writing–original draft. HN, JA, EMP, and JPG: writing–review and editing. All authors have read and agreed to the published version of the manuscript.

## Conflict of Interest

The authors declare that the research was conducted in the absence of any commercial or financial relationships that could be construed as a potential conflict of interest.

## Publisher's Note

All claims expressed in this article are solely those of the authors and do not necessarily represent those of their affiliated organizations, or those of the publisher, the editors and the reviewers. Any product that may be evaluated in this article, or claim that may be made by its manufacturer, is not guaranteed or endorsed by the publisher.
